# Antibacterial and Photocatalytic Activities of LDH-Based Sorbents of Different Compositions

**DOI:** 10.3390/microorganisms11041045

**Published:** 2023-04-16

**Authors:** Anna Maria Cardinale, Stefano Alberti, Andrea Pietro Reverberi, Michelina Catauro, Nicolò Ghibaudo, Marco Fortunato

**Affiliations:** 1 DCCI, Department of Chemistry and Industrial Chemistry, Università degli Studi di Genova, Via Dodecaneso 31, 16146 Genova, Italy; stefano.alberti@unige.it (S.A.); andrea.reverberi@unige.it (A.P.R.); nicolo.ghibaudo@edu.unige.it (N.G.);; 2 Department of Engineering, University of Campania “Luigi Vanvitelli”, Via Roma 29, 81031 Aversa, Italy; michelina.catauro@unicampania.it

**Keywords:** ZnAl LDH, chromium (VI) removal, heterogeneous photocatalysis, water remediation, pollutants degradation, antimicrobial behaviour

## Abstract

Layered double hydroxides (LDHs) play a fundamental role in the processes for the abatement of pollutants in water, with reference to heavy metal decontamination. The research on the topic is multiobjective target oriented, aiming at combining environmental remediation with the possibility of reusing a sorbent as many times as possible, turning it into a renewable resource. In this study, the antibacterial and catalytic properties of a ZnAl-SO_4_ LDH and its resulting product after being subjected to a Cr(VI) remediation process are compared. Both solid substrates have also been tested after undergoing a thermal annealing process. The sorbent (previously described and tested for remediation) has been investigated for its antibacterial activity in view of further surgery and drug delivery applications. Finally, its photocatalytic properties have been experimentally tested in the degradation of a model pollutant, i.e., Methyl Orange (MO), under solar-simulated light. Identifying the best recycling strategy for these materials requires an accurate knowledge of their physicochemical properties. The results show that both the antimicrobial activity and the photocatalytic performance may considerably improve after thermal annealing.

## 1. Introduction

Layered double hydroxides (LDHs) represent a wide ensemble of compounds currently in the hotspot of scientific research owing to their extreme versatility in many applications. From a compositional point of view, they are hydroxides of divalent and trivalent cations of general expression [M(II)_1−x_M(III)_x_(OH)_2_]^x+^[(A^n−^)_x/n_ mH_2_O], and they are characterized by a double positively charged brucite-layer, separated by a negatively charged interlayer where host anions are present and replaceable, thus making them useful as anionic exchangers generally known as “anionic clays”.

Initially used as materials with excellent sorption properties, they proved to be particularly suitable in the adsorption of both anions [1] and cations, with obviously different physicochemical mechanisms according to the charge of the species to be retained. In the first case, the M(II)/M(III) molar ratio was one of the most important parameters tuning the anion affinity [2], which has been widely tested in experimental studies concerning halogenated anions of type X^−^ (X = F, Cl, Br, I), related oxoanions such as XO_3_^−^ (X = Cl, Br, I) and other specific anions having well-established toxic properties towards the environment and even carcinogenic activity for mammalians [3,4,5], as thoroughly reviewed by [6].

The sorption efficiency of LDHs towards inorganic cations [7] was amply tested in environmental remediation thanks to their selective affinity for polluting species such as Pb(II), Hg(II), Cd(II) and even radioactive isotopes as by-products deriving from nuclear power plants producing liquid radioactive wastes [8]. In this context, the review paper by [9] is a very exhaustive survey, giving a very wide picture of LDHs of different compositions as adsorbing substrates. They analyzed the mechanisms of heavy metals capture, where chemical precipitation [10], cation complexation and electrostatic attraction [11] at the LDH surface are the most important phenomena governing the abatement of such noxious species. In many cases of heavy metal decontamination, LDH adsorption performances proved to be competitive, in both economic and management terms, with those offered by the corresponding biosorption processes [12].

In addition to their affinity to both cations and anions, LDHs as sorbents also have a very interesting peculiarity: they proved to be likewise efficient in the abatement of organic polluting compounds, including dyes, pesticides [13] and pharmaceuticals [14], the latter representing a crucial subset of emerging contaminants affecting developing countries, where a high concentration of antibiotics have been detected in soils and water [15]. In the comprehensive survey presented by [16] about correlations between the structure and sorption efficiency of LDHs, authors pointed out that the main processes governing the adsorption of organic pollutants are based on outer surface sorption and interlayer anion exchange, having a basic role in the presence of anionic molecules like Methyl Orange (MO), which can be assumed as a sort of prototypical dye in the current literature on the topic. Interestingly, they stressed that there is still a lack of investigation concerning the role of interlayer orientation in determining the LDHs’ sorption performances, which can be dramatically low in the presence of organic molecules with a predominance of cationic groups [17].

The versatility of LDH use is proven by their results as efficient catalysts and photocatalysts with a broad spectrum of structural and compositional variants [18]. In the first case, the LDH served as a substrate for host molecules already employed as homogeneous catalysts. With such a strategy, the drawbacks typical of homogeneous catalysis can be overcome, with benefits in terms of thermal stability and product separation [19]. On that note, the production of valuable chemicals by CO_2_ conversion [20], such as CO_2_ hydrogenation to methanol and hydrocarbons, by LDH-based catalysts are well-known and challenging fields of research, in line with the recent protocols of green energy and sustainable manufacturing, where the minimization of greenhouse gases is a pressing target [21]. In this context, against a large amount of experimental data, there is a growing and pressing need for accurate modeling of reaction kinetics, which does not seem to have been developed with the same trend, as pointed out by some investigators, also considering that the best opportunities for a safer process development are connected to the early-stage development [22]. In the second case, the more recent findings in LDH-driven photocatalysis have ushered a new phase in LDH research and applications, as the possibility of compositional and structural variants in the choice of LDH photocatalysts is even greater than the one typical of the conventional LDH-based catalysts [23]. In the line of research focused on LDH-assisted photocatalysis, two trends can be distinguished. The first one is focused on pristine LDH materials, which can be considered as homogeneous photocatalysts whose efficiency can be tuned by varying both cation composition and M(II)/M(III) ratio in the brucite layers, as adopted in CO_2_ photoreduction of the first generation [24,25]. However, the efficiency of genuine LDH photocatalysts suffers from some serious drawbacks related both to a limited specific surface area and to an intrinsically small diffusivity of charge carriers to the surface, thus acting as rate-limiting steps dramatically affecting the overall kinetics of photocatalytic reactions. The advent of surface nanotechnology allowed these disadvantages to be overcome by new synthesis techniques to produce monolayered or ultrathin-layered LDH photocatalysts with promising results in artificial photosynthesis [26], nitrogen oxide conversion to nitrate ion [27] and NH_3_ synthesis by N_2_ photoreduction [28]. The second trend concerns the realization of composite and hybrid photocatalysts, where an LDH-based structure is coupled with different co-catalysts, whose heterojunction has a basic role in reducing electron-hole recombination with a significant increase in chemical process yield, independently of the specific reaction [29]. Likewise, the negative effect of a large bandgap, making the use of UV light almost mandatory, can now be considerably damped, thus making composite LDH photocatalysts useful in water splitting by visible light [30]. These hybrid and composite LDH-based mixtures allowed the realization of sensors for trace-level analysis of organic and inorganic pollutants [31], with important applications in many fields of analytical chemistry [32], including optical sensing [33].

The recent use of LDHs as antibacterial substrates is further recent proof of LDH versatility in their multipurpose applications [34,35]. For example, Ref. [36] synthesized LDH-organic composites by intercalation of benzoic acid derivatives between Zn-Al LDH interlayers using an anionic exchange technique. The antibacterial properties of the as-prepared composite were tested against colonies of Gram-positive and negative pathogens.

The present study can be traced back to this context in that a ZnAl-SO_4_ LDH substrate has been tested for photocatalytic and antibacterial properties in four different structural and compositional variants. The novel aspects of this study consist of applying the three Rs (Reduce, Reuse, Recycle) philosophy to an eco-friendly compound primarily useful in the remediation field. The paper is divided as follows. In Section 2, the synthesis process is described, together with the characterization techniques. In Section 3, the results are presented both for the photocatalytic activity with respect to the abatement of a selected benchmark target molecule and for the antibacterial activity against standard pathogens. In Section 4, the conclusions are drawn, and a future research line is traced.

## 2. Materials and Methods

### 2.1. Reagents for Synthesis, Photocatalytic and Antimicrobial Tests

Aluminum sulfate (Al_2_(SO_4_)_3_·9H_2_O, 99%, VWR Chemicals, Leuven, Belgium), zinc nitrate (Zn(NO_3_)_2_ 7H_2_O, 99%, VWR Chemicals, Leuven, Belgium), sodium hydroxide (NaOH, >97%, Merck, Milano, Italy), potassium chromate (K_2_CrO_4_, >99%, Sigma Aldrich, St. Louis, MO, USA), Methyl Orange (MO, C_14_H_14_N_3_NaO_3_S, >98%, Merck, Darmstadt, Germany) were used as purchased. For chromium binding experiments, the latter reagent was used to prepare calibrated solutions of 1000 mg/L K_2_CrO_4_ in water. All reactions were carried out in aqueous solvent, using deionized water produced by an ionic exchange unit (M3/M6 Chemical Bürger s.a.s, Genova, Italy).

The assessment of antimicrobial activity was carried out using *Escherichia coli* strains (ATCC25922), *Staphilococcus aureus* strains (ATCC25923), TBX Medium (Tryptone Bile X-Gluc), Baird Parker Agar Base, Egg Yolk Tellurite emulsion 20% and isotonic sodium chloride solution (NaCl, 0.9% in water). All reagents for antimicrobial tests were purchased from Liofilchem S.r.l., Teramo, Italy.

### 2.2. LDH Synthesis

The synthesis of the ZnAl sulfate–LDH, with a molar ratio Zn/Al = 3, has been carried out following a standard procedure [7], relying upon a coprecipitation reaction followed by an aging process under heating. In all experiments, the deionized water used in each synthesis step was decarbonated by boiling and further stripping by argon to minimize the presence of CO_2_ in the reaction environment. For the same reason, the global process was carried out under argon atmosphere to avoid contamination by CO_3_^2−^ anions in the interlayer structure, where only SO_4_^2−^ anions should be present, to maximize the overall LDH performances.

The two sulfates, previously weighted and mixed according to the Zn/Al molar ratio, were dissolved in 150 mL of boiled deionized water and kept at rest in a beaker under argon atmosphere. A three-necked flask, where 200 mL of deionized water was put and kept in agitation by a magnetic stirrer, served as a reactor equipped by an electrode for pH measurement. The pH value was monitored by means of a pH-meter pH/ORP/ISE Single Channel Benchtop Meter-HI3221 (HANNA Instruments, Woonsocket, RI, USA).

Afterward, the aqueous phase carrying the mixed sulfates was added dropwise into the flask, keeping the pH constantly fixed at 8 ± 0.5 by continuously adding NaOH solution dropwise. Sodium hydroxide not only acted as a reagent, but it allowed an online correction of the holdup pH. For this reason, it was separately prepared in solutions having growing concentrations up to 2 m to ensure a timely intervention if required. The whole process was carried out under magnetic stirring at approximately 900 r.p.m.

Once the mixing of the reagents was complete, the solution embedding the as-formed solid phase was transferred into a dark sealed vessel, and the suspension was aged in a stove at 50 °C for a week. This relatively long-standing time was necessary so that the solid could attain a stable crystalline structure. Afterward, the as-formed crystalline LDH was separated by filtration, repeatedly washed with water and finally dried in a static oven at 50 °C for 24 h. The yield of the processes is about 80%.

The product obtained according to this synthesis procedure underwent different and mutually exclusive after-synthesis processes. From this point on, for the sake of clarity, the three different solid phases tested in the remainder of this study will be named ZnAl-SO_4_ LDH, ZnAl-MMO (mixed metals oxide), ZnAl-CrO_4_ LDH and ZnAl-MMO-CrO_4_ according to the following scheme:

- ZnAl-SO_4_ LDH is the pristine LDH phase, with no after-synthesis process.
- ZnAl-MMO is the phase obtained by the ZnAl-SO_4_ LDH annealing at 450 °C for 5 h. This process transforms the pristine LDH structure in ZnAl mixed oxide, whose structure maintains a certain disorder degree.
- ZnAl-CrO_4_ LDH is the phase resulting from Cr(VI) adsorption, up to saturation, on a pristine ZnAl-SO_4_ LDH sorbent, according to a protocol described in a previous study [37].
- ZnAl-MMO-CrO_4_ is the mixed oxide from the annealing process saturated with Cr(VI).

### 2.3. Characterization Techniques

All the compounds, after being prepared, were characterized by means of several techniques: Inductively Coupled Plasma-Atomic Emission Spectroscopy (ICP-AES), X-ray powder diffraction (XRPD), Field Emission Scanning Electron Microscope (FE-SEM) and Fourier Transform Infrared Spectroscopy (FT-IR). The ICP-AES measurements were performed using an axially-viewed Varian (Springvale, Australia) Vista PRO. The sample introduction system consisted of a glass concentric K-style pneumatic nebulizer (Varian) jointed to a glass cyclonic spray chamber (Varian). To compensate for non-spectral interferences, the online internal standardization (4 μg mL^−1^ Lu standard solution) was applied. XRPD patterns were collected to identify the eventual crystalline phase using X’Pert MPD (Philips, Almelo, Netherland) X-ray powder diffractometers equipped with a Cu anticathode with Cu Kα1 radiation (λ = 1.5406 Å), the samples were prepared by grinding them in an agate mortar. The patterns were collected between 10° and 100° 2θ with a step of 0.001° and measuring time of 50 s/step.

The indexing of the obtained diffraction data was performed by comparison of signals with those available in the literature [37], and the lattice parameters of the phases were calculated using the LATCON program [38]. A ZEISS SUPRA 40 V model of the field emission scanning electron microscope (FE-SEM) was used, where the sample was analyzed by applying an acceleration voltage of 5 kV for 50 s. FT-IR spectra were collected in the usual wavelength range from 4000 to 600 cm^−1^ using a Spectrum 65 FT-IR Spectrometer (PerkinElmer, Waltham, MA, USA) equipped with a KBr beam-splitter and a DTGS detector by using an ATR accessory with a diamond crystal.

### 2.4. Antimicrobial Activity Tests

The following procedure has been applied to evaluate the antimicrobial activity of ZnAl-SO4 LDH and ZnAl-MMO. The bacteria used for this test were both Gram-negative (*Escherichia coli*) and Gram-positive (*Staphylococcus aureus*) incubated in the presence and absence of the sample.

Prior to the analysis, the sample powder was compressed into two small disks of 150 mg and 300 mg, respectively. These disks were then sterilized under UV light for 1 h.

TBX Medium (Tryptone Bile X-Gluc) and Baird Parker Agar Base were prepared by dissolving each nutrient in autoclaved water. Each bacterial media was sterilized at 120 °C for 15 min and slowly cooled. TBX was poured directly into Petri dishes at 50 °C, while the Baird Paker Agar was cooled up to 50 °C and enriched with Egg Yolk Tellurite emulsion 20%.

The antimicrobial properties were evaluated using the Kirby–Bauer method [39,40]. The pellets of the bacterial strains were dissolved in isotonic saline water (0.9% NaCl) and diluted, obtaining bacterial suspensions of 105 CFU/mL, which were plated on the respective solid agar media. *E. coli* was plated on TBX Medium and *S. aureus* on Baird Parker Agar. Before the incubation time of bacteria, the sample disks of 150 and 300 mg were placed in the middle of Petri dishes. *E. coli* was incubated at 44 °C for 24 h, while *S. aureus* was incubated at 36 °C for 24 h. To evaluate the antimicrobial activity, after the incubation time, the diameters of the inhibition halos (IHDs) were calculated. For each sample, to determine the mean Standard Deviation, four measures of the diameter were taken.

### 2.5. Photocatalytic Experiments Setup

Photocatalytic experiments were performed to investigate the possibility of reusing the tested ZnAl-SO_4_ LDH materials, before and after the Cr(VI) remediation process as well as before and after a thermal treatment, for environmental purposes. Since the capability of some layered double hydroxide materials to act as heterogeneous photocatalysts are reported in the literature, it was decided to test the photocatalytic performances of the substrates listed at the end of Section 2.2 (Table 1) for water purification from emerging contaminants [41,42,43,44].

A volume of 50 mL of 0.01 g L^−1^ (10 ppm) Methyl Orange (MO) aqueous solution (pH = 5.5, T = 293 K) was employed as a model pollutant: powdered samples were dispersed within the solution at a concentration equal to 0.25 mg mL^−1^ and subjected to solar simulated light irradiation with an OSRAM Ultra-Vitalux lamp (300 W) placed 20 cm above the solutions. MO dye, being a typically recalcitrant pollutant, was chosen as a test molecule for assessing photocatalysts’ activity. Solar lamp’s irradiance is reported to be 1.1 W m^−2^ in the UV-B range, 7.3 W m^−2^ in the UV-A range and 29.7 W m^−2^ in the Vis range. Experiments were performed for a total of 120 min while aliquots were withdrawn every 15 min. The percentage degradation Deg (%) of the dye was measured according to the following equation:

Deg (%) = (C_0_ − C_t_)/C_0_ × 100 (1)

A blank experiment was performed to study the stability of MO under irradiation, in the same conditions exploited for the photocatalytic tests but without the dispersed material. Further, to investigate the adsorption/desorption equilibrium between tested materials and MO, thus the contribution of the photocatalytic process, the same experiments were performed in dark conditions, without lamp irradiation. Adsorption kinetics were investigated for tested samples by modeling both pseudo-first-order and pseudo-second-order, according to [45,46,47,48]. Prior to the analysis, aliquots withdrawn from the solution were subjected to 13,200 rpm centrifugation for 10 min to remove suspended materials, and eventually, MO concentration was quantified using a UV-Vis LAMBDA 35 (Perkin Elmer, Whaltam, MA, USA) spectrophotometer, monitoring MO maximum absorbance (λ = 463 nm). Each sample was tested in duplicate.

## 3. Results and Discussion

**Figure 1:**
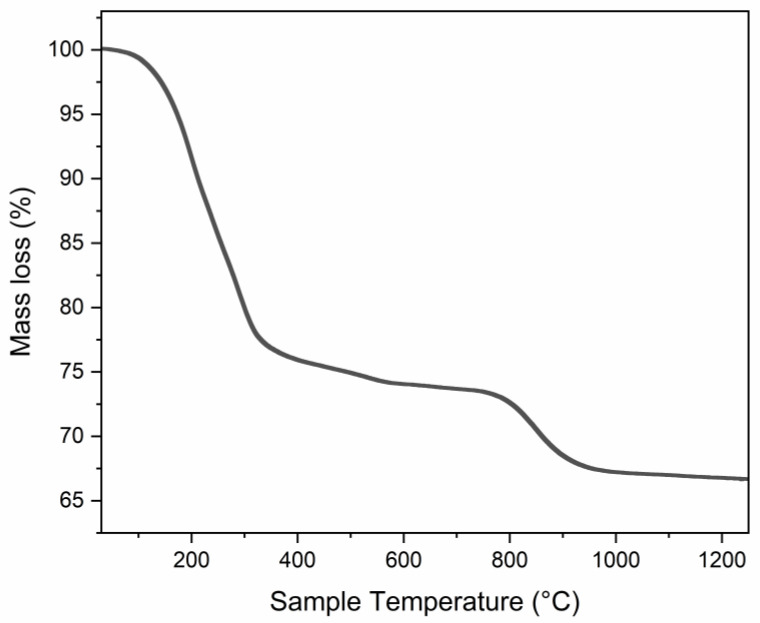
TGA curve for the ZnAl-SO_4_ LDH from [7].

The pristine LDH compound has been characterized, and the results confirm the structure demonstrated in [7]; nevertheless, the TGA curve is reported to explain the annealing temperature chosen. The thermogravimetric pattern for ZnAl-SO_4_ LDH, observable in Figure 1, shows three main mass losses, with a global mass decrease of about 33 mass%. The first thermal effect, at about 100 °C, is due to the humidity, while the second one, occurring in a temperature range of 200–550 °C corresponds to the loss of crystallization water and probably to a release of CO_2_ entrapped in the structure (as seen in the FTIR spectrum). The last thermal effect, starting at about 710 °C, is attributable to SO_2_ gas evolution.

**Figure 2:**
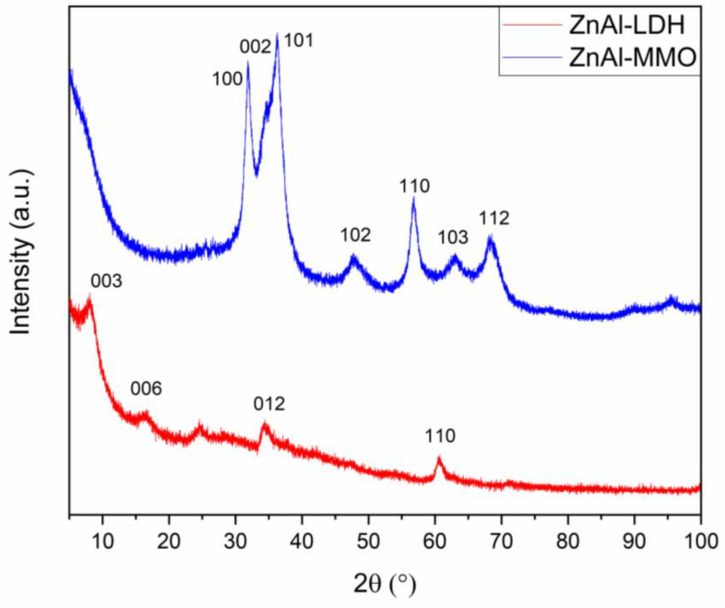
PXRD pattern for the pristine LDH compound (red line) and the ZnAl-MMO (blu line).

Owing to the thermal behavior on heating and according to the literature [49,50,51], the pristine LDH has been annealed at 450 °C for 5 h to obtain the ZnAl-MMO. In Figure 2, the PXRD patterns for both pristine LDH and ZnAl-MMO resulting from annealing are reported, together with the main reflection indexes. The structure of the mixed oxide, belonging to the space group 186, hP4-ZnO type, can be identified in the ZnAl-MMO.

By comparing the two PXRD patterns, it is highlighted a change in the structure and a transformation of the layered double hydroxide in a Zn Al mixed oxide, which retains a turbostratic structure, as shown by the asymmetric feature of the peaks.

**Figure 3:**
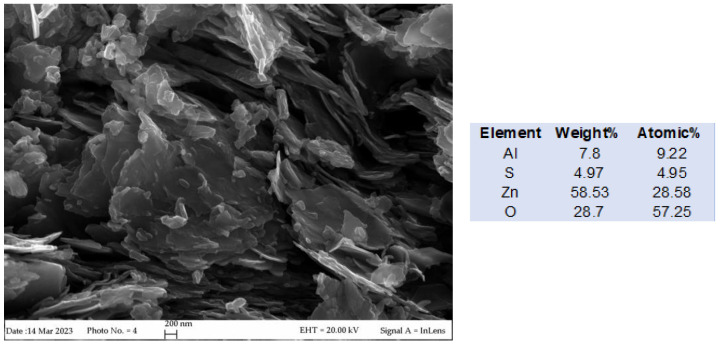
FESEM image of the ZnAl-MMO morphology and composition.

Morphology and composition of the annealed LDH (ZnAl-MMO) have been investigated by FESEM analysis, and the results are reported in Figure 3.

The ZnAl-MMO compound retains the typical lamellar feature, and the EDXS analysis confirms the Zn/Al ratio also in the mixed oxide.

Both the pristine compound and the annealed one have been tested for their antimicrobial behavior, while the samples containing a high concentration of Cr(VI) anion were not subjected to an analogous test.

**Figure 4:**
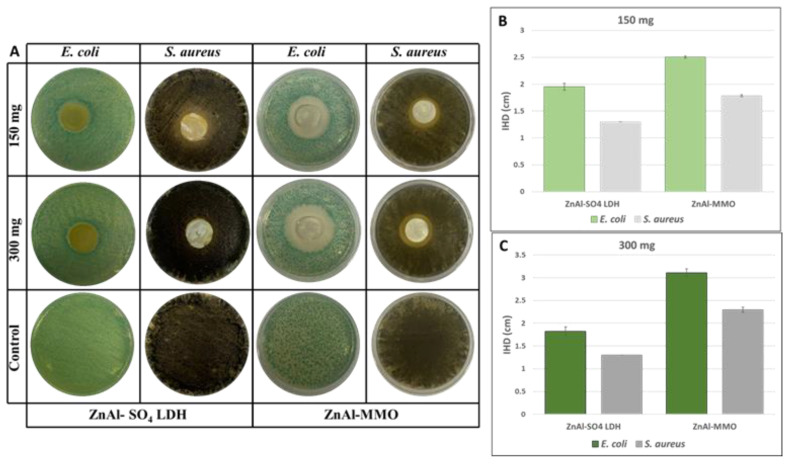
(**A**) *E. coli* and *S. aureus* images obtained after incubation in the presence and absence of ZnAlSO_4_-LDH and ZnAl-MMO. Inhibition halo diameters (IDHs) of ZnAlSO_4_-LDH and ZnAl-MMO samples obtained with (**B**) 150 mg and (**C**) 300 mg.

Figure 4 shows the results of the antimicrobial analysis, and Figure 4B,C reports the values of the inhibition halos.

By simple inspection of Figure 4A, the ZnAl-SO_4_-LDH, regardless of the amount analyzed, does not have significant antimicrobial activity against both *E. coli* and *S. aureus* bacteria. However, the activity against *E. coli* was slightly higher than the one against *S. aureus*, whose IHDs were equal to the diameters of the sample disks (Figure 4B,C, 1.3 cm), as no growths on them were observed. The average inhibition halos of untreated ZnAl-SO_4_-LDH against *E. coli* were 1.95 and 1.79 cm for the 150 and 300 mg disks (Figure 4B,C), respectively. Before thermal treatment, the inhibition power does not seem to be related to the quantity assayed; in fact, the inhibition halo of 150 mg disk is quite equal to the one obtained with 300 mg disk. An explanation could be related to the fact that a larger quantity of material can more easily form complex, leading to the partial loss of the functional groups responsible for the antibacterial activity [52,53]. However, the antimicrobial activity of ZnAl-SO_4_ LDH is also reported in the literature [54]. After thermal treatment at 450 °C, the antimicrobial activity seems to be enhanced. Indeed, both bacteria were inhibited by the ZnAl-MMO compound. In particular, the antimicrobial effects against *E. coli* (2.5 and 3.1 cm) were greater than those observed against *S. aureus* (1.8 and 2.3 cm). Moreover, the treatment also affects the dose-dependent activity. Indeed, an increase in the sample amount resulted in an increase in the IHDs. This is in accordance with the finding that Zn^2+^, released from ZnO particles interacting with the bacterial media, may have an important antimicrobial activity against *Escherichia coli*, *Staphylococcus aureus*, and *Pseudomonas aeruginosa* [55].

**Figure 5:**
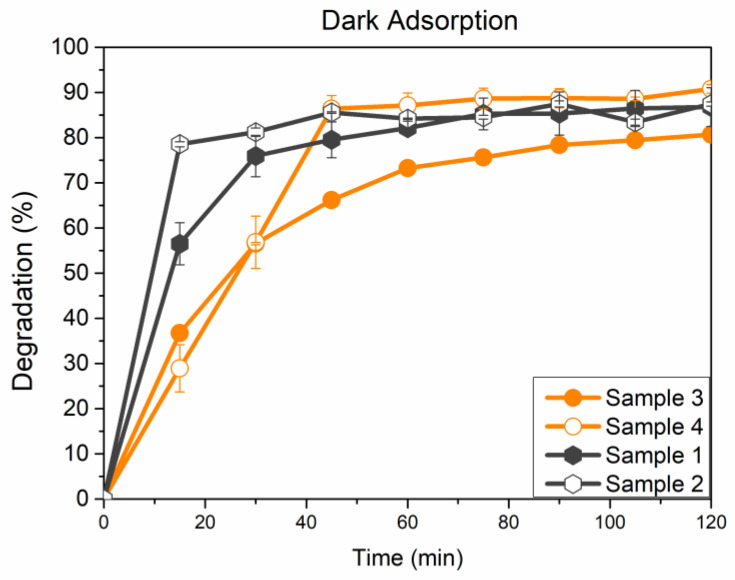
MO percentage degradation (calculated according to Equation (1)) for investigated samples in dark condition.

The results for the catalytic activity in the dark, thus concerning only adsorption experiments, are reported in Figure 5, while the relevant kinetics, modeled as pseudo-first and pseudo-second-order rate laws, are reported in Table 2.

All the experiments performed in dark conditions are reported in Figure 5. All tested materials show a quite pronounced tendency to interact with the MO dye, specifically showing surface adsorption, which subtracts the dye from the solution. Samples show similar behavior and reach an average final value of 85% in MO degradation (%) after 120 min in all cases. Instead, considering the first 15 min, some differences can be observed: Sample 3 and Sample 4 are slower in the MO adsorption, probably because they already absorbed chromate ions within their structure. Sample 1 and Sample 2, at the same time, double the percentage of MO adsorbed with respect to Samples 3 and 4. The calcination process has likely modified the materials’ features, as in both cases, a slight increase in the MO adsorbed concentration can be noticed. Finally, all samples attained an adsorption–desorption equilibrium in the first hour of the experiment, ending with a high value of dye adsorption. According to the observed results, the experimental data were better fitted by a pseudo-second-order kinetics rate law, as shown by R^2^ values, which are quite high (>0.99) when compared to pseudo-first-order ones. Furthermore, the values of q_e_ computed using a pseudo-second-order model are closer to the corresponding experimental values than the ones obtained by adopting a first-order kinetics model.

**Figure 6:**
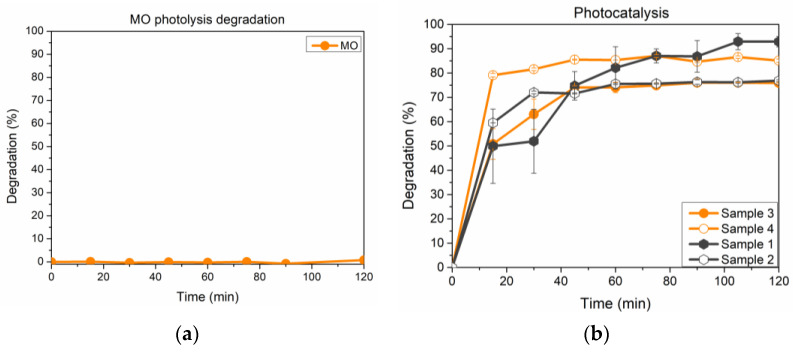
(**a**) MO percentage degradation due to photolysis (MO stability under irradiation); (**b**) MO percentage degradation (calculated according to Equation (1)) for investigated samples under solar simulated light.

To try out the heterogeneous photocatalytic performance of the tested materials, the same experiments were performed under simulated solar light exposure, and results are reported in Figure 6b, while Figure 6a reports the results concerning MO stability under irradiation.

**Figure 7:**
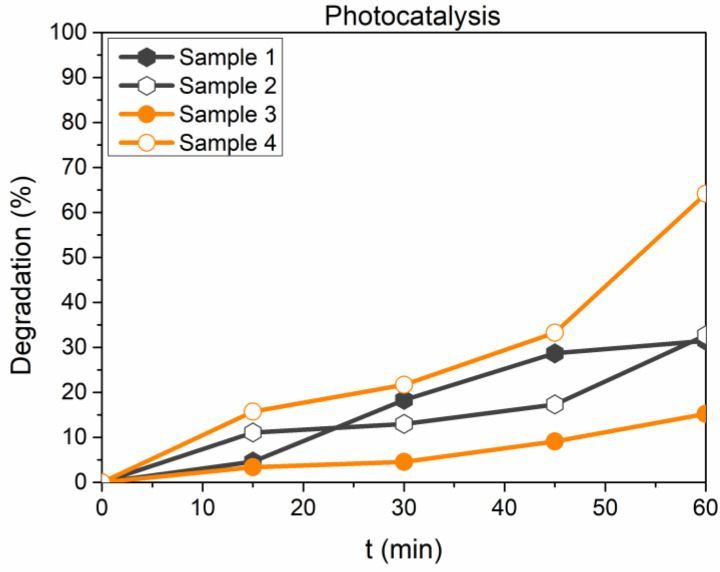
MO percentage degradation (calculated according to Equation (1)) for investigated samples.

Methyl Orange itself proved to be stable under solar light exposure, as no change in the concentration was detected. Unfortunately, samples still show a marked tendency to adsorb MO dye in a way that makes it difficult to ascertain the effect of photocatalysis. To clarify this trend, another test run was carried out at the same experimental conditions before irradiation, with samples left under stirring in the dark for 60 min. Namely, Figure 6 shows that all solid substrates interact with MO in the first 60 min, after which the concentration remains stable. The photocatalytic test is then carried out for an additional 60 min, and the results are reported in Figure 7.

As a general conclusion, it seems that Samples 1 and 2 strongly interact with the dye in dark conditions, and this implies that adsorption occurs on the sample’s surface; the same interaction can be observed for Samples 3 and 4, even though the rate of adsorption is slower. After an initial adsorption phase, when solar light irradiation is switched on, Sample 4 turns out to be the most performing, as it can reach a final abatement of 60% only due to irradiation. Furthermore, the dye degradation obtained by the same sample without thermal treatment stands at around 15%, proving that the thermal treatment strongly influences the photocatalytic trend. Both samples 1 and 2 achieve a final abatement degree of 30% without any changes relatable to the thermal process. This could be reasonably ascribed to the tendency of the material to adsorb chemical species present in the liquid phase: whenever the samples are pre-treated as adsorbers, the photocatalytic activity is predominant over the adsorption process. On the opposite, bare samples have a significant tendency to adsorb the dye instead of degrading it.

## 4. Conclusions

In the present study, ZnAl-SO_4_ LDH substrates and other solid matrices derived from them have been investigated for their photocatalytic and antimicrobial properties. The most important results can be summarized in the following points:

- The antimicrobial activity increases after heat treatment is performed, which moreover influences the effect of dose dependence. This result may open interesting new scenarios in drug delivery field applications.
- The photocatalytic activity of the investigated compounds is well described by pseudo-second-order kinetics, despite the calculated values of *q_e_*, for both pseudo-first- and pseudo-second-order kinetics are not markedly different.
- The overall performances are affected by adsorption phenomena, and this result is more pronounced for the two compounds without the chromate anion. The results seem to suggest that the presence of chromate ions within the structure may influence the photocatalytic activities of these materials, thus creating new opportunities in recycling exhaust LDH, having adsorbed contaminants like Cr(VI), by turning it into a photocatalyst of promising performances. On the other hand, an increase of catalytic activity following the adsorption of Cr(VI) would hamper the use of this substrate for medical or drug delivery purposes, owing to the well-known toxicity of the adsorbed anion.

## Figures and Tables

**Table 1:** Scheme of the substrates used in the present tests concerning photocatalytic abatement of contaminants.

Sample n.	Composition
1	ZnAl-SO_4_ LDH
2	ZnAl-MMO
3	ZnAl-CrO_4_ LDH
4	ZnAl-MMO-CrO_4_

**Table 2:** Adsorption kinetic model rate constants for MO on investigated samples.

**Sample n.**	**Pseudo-First-Order** **eq.**	**q_e_ (exp.)** **(mg g^−1^)**	**q_e_ (calc.)** **(mg g^−1^)**	**k_1_** **(min^−1^)**	**R^2^**
1	y = −0.0038x – 1.9017	0.0378	0.0125	0.0087	0.7974
2	y = −0.0014x – 2.1001	0.0373	0.0079	0.0032	0.575
3	y = −0.0043x – 1.7303	0.0356	0.0186	0.0099	0.8928
4	y = −0.0067x – 1.7674	0.0350	0.0170	0.0154	0.6993
**Sample n.**	**Pseudo-Second-Order eq.**	**q_e_ (exp.)** **(mg g^−1^)**	**q_e_ (calc.)** **(mg g^−1^)**	**k_2_** **(g mg^−1^ min^−1^)**	**R^2^**
1	y = 30.877x + 58.531	0.0378	0.0355	16.2886	0.9992
2	y = 31.732x + 20.434	0.0373	0.0288	49.2766	0.9985
3	y = 34.666x + 169.79	0.0356	0.0315	7.0777	0.9955
4	y = 28.122x + 104.29	0.0350	0.0323	7.5831	0.9944

## Data Availability

Not appliable.
